# Metabolic Profiling Framework for Discovery of Candidate Diagnostic Markers of Malaria

**DOI:** 10.1038/srep02769

**Published:** 2013-09-26

**Authors:** Lucienne Tritten, Jennifer Keiser, Markus Godejohann, Jürg Utzinger, Mireille Vargas, Olaf Beckonert, Elaine Holmes, Jasmina Saric

**Affiliations:** 1 Department of Medical Parasitology and Infection Biology, Swiss Tropical and Public Health Institute, CH-4002 Basel, Switzerland; 2 University of Basel, CH-4003 Basel, Switzerland; 3 Bruker BioSpin GmbH, 76287 Rheinstetten, Germany; 4 Department of Epidemiology and Public Health, Swiss Tropical and Public Health Institute, CH-4002 Basel, Switzerland; 5 Section of Computational and Systems Medicine, Department of Surgery and Cancer, Faculty of Medicine, Imperial College London, South Kensington, London, SW7 2AZ, United Kingdom

## Abstract

Despite immense efforts to combat malaria in tropical and sub-tropical regions, the potency of this vector-borne disease and its status as a major driver of morbidity and mortality remain undisputed. We develop an analytical pipeline for characterizing *Plasmodium* infection in a mouse model and identify candidate urinary biomarkers that may present alternatives to immune-based diagnostic tools. We employ ^1^H nuclear magnetic resonance (NMR) profiling followed by multivariate modeling to discover diagnostic spectral regions. Identification of chemical structures is then made on the basis of statistical spectroscopy, multinuclear NMR, and entrapment of candidates by iterative liquid chromatography (LC) and mass spectrometry (MS). We identify two urinary metabolites (i) 4-amino-1-[3-hydroxy-5-(hydroxymethyl)-2,3-dihydrofuran-2-yl]pyrimidin-2(1H)-one, (ii) 2-amino-4-({[5-(4-amino-2-oxopyrimidin-1(2H)-yl)-4-hydroxy-4,5-dihydrofuran-2-yl]methyl}sulfanyl)butanoic acid that were detected only in *Plasmodium berghei*-infected mice. These metabolites have not been described in the mammalian or parasite metabolism to date. This analytical pipeline could be employed in prospecting for infection biomarkers in human populations.

P*lasmodium falciparum* is the most deadly human parasite, inflicting the greatest mortality and global burden of all parasitic diseases^1^. In 2010, *P. falciparum* accounted for approximately 216 million clinical cases and more than 600,000 deaths, mostly amongst children below the age of 5 years in sub-Saharan Africa. Antigen-detecting rapid diagnostic tests (RDTs) for malaria have mushroomed in the last few years and have undergone rigorous testing. The development and large-scale deployment of RDTs as point-of-care tests has revolutionized malaria management. Clinical sensitivity, however, still leaves ground for improvement, particularly in cases of low parasitemia^2^.

An additional complication is that in many tropical and sub-tropical regions, people are concurrently infected with *Plasmodium* and parasitic worms (helminths), particularly in remote rural areas. One quarter of all school-aged children on the African continent (~45 million) are likely to harbor concomitant infections with *P. falciparum* and hookworm^3^, explained by the high degree of geographic overlap of the parasites. One obvious question is to what extent a parasitic worm infection affects diagnosis, immune response, clinical manifestation, and prognosis of malaria^4^,^5^.

Metabolic profiling has been used to gain knowledge about host-parasite interactions and to discover infection-related metabolite patterns. Thus far, the majority of exploratory work has been conducted in rodent models wherein each host-parasite model is represented by an infection-specific fingerprint in urine and/or plasma^6^,^7^,^8^,^9^,^10^.

Despite the widely assumed universality of eukaryotic metabolism, some class- or species-specific adaptions exist in parasites, such as the trypanothione system in kinetoplasts (*Leishmania* spp. and *Trypanosoma* spp.) that replaces glutathione-mediated detoxification of reactive oxygen species^11^. Compounds of exogenous origin introduced into mammalian biofluids, represent ideal diagnostic biomarkers due to the specificity of the metabolite. Given that a comprehensive human map of metabolism is still limited to date, and that between the human metabolome database (HMDB) versions 2.0 and 3.0, the number of endogenous metabolites increased several-fold^12^, it is realistic to assume that a significant pool of potential unexplored metabolic biomarkers exists, both in the mammalian and in the parasitic system.

In the present study, the malaria parasite *P. berghei* and the intestinal nematode *Heligmosomoides bakeri*, commonly utilized as a model for hookworm infection^13^ are employed to establish murine models of malaria with and without concurrent helminth infection. An analytical pipeline is developed to structurally identify key diagnostic metabolic components driving the urinary infection signatures.

## Results

### Experimental setup and rationale

**Figure 1:**
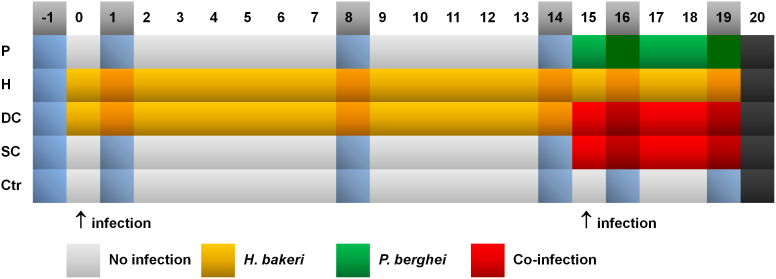
Experimental design of the infection models. Urine and plasma samples are collected from all mice (5 groups, n = 8) one day before and 1, 8, 14, 16, and 19 days after the start of the experiment (day 0). The darker cells represent sampling days, the arrows represent infection timepoints. P: *P. berghei* only; H: *H. bakeri* only; DC: delayed co-infection; SC: simultaneous co-infection; Ctr: uninfected control.

To investigate the metabolic effects of a malaria-hookworm co-infection in the murine host in a laboratory-controlled experiment, and to identify candidate biomarkers that are predictive for *P. berghei* during single or co-infection, we randomly divided mice into five groups, (i) single infection with *P. berghei* (group P); (ii) single infection with the mouse hookworm *H. bakeri* (group H); (iii) simultaneous co-infection with both parasites (group SC); (iv) infection with *H. bakeri* prior to infection with *P. berghei* (group DC); and (v) uninfected (group Ctr) (Fig. 1). The experiment was run for 20 days in order to establish a chronic helminth infection prior to malaria co-infection. Groups H and DC were infected with *H. bakeri* on day 0 and groups P and DC were inoculated with *P. berghei* on day 15. Group SC was simultaneously infected with both parasites on day 15. Plasma and urine samples were collected one day pre-infection and five times during the course of infection up to day 19 post-infection.

### Identification of candidate urinary biomarkers of *P. berghei*

Both *P. berghei* and the co-infection groups manifest systematic changes in the urinary metabolite profiles, as determined by orthogonal partial least squares discriminant analysis (O-PLS-DA) of the NMR data. Pipecolic acid and three initially unknown metabolites (UK1, UK2, and UK3), are present in the *P. berghei*-infected groups (Table 1) on day 16 (1 day postinfection with *P. berghei*). These four metabolites are not detectable in the control group (Ctr) or in mice bearing a single *H. bakeri* infection (H). The identity of pipecolic acid has previously been confirmed by NMR by spiking of the reference compound into urine from an infected animal^9^,^14^. Pipecolic acid is a known component of urine but is typically not detected in NMR urine profiles due to its low concentration.

UK1 and UK2 are structurally identified using ultra high performance liquid chromatography-time of flight-tandem mass spectrometry (UPLC-TOF-MS/MS) and liquid chromatography-NMR/time of flight mass spectrometry (LC-NMR/TOF-MS). Accurate MS, MS/MS, 1-dimensional (1D) and 2-dimensional (2D) NMR experiments were conducted to elucidate the structure of UK2. The MS/MS spectrum shows a neutral loss of cytosine (C_4_H_5_N_3_O) from the parent ion at m/z 343.1083 corresponding to a molecular formula C_13_H_19_N_4_O_5_S (Fig. S1). The resulting fragment with an m/z of 232.0645 corresponds to the even electron ion with the molecular formula C_9_H_14_NO_4_S (didehydro ribose-homocysteine moiety). A second fragmentation yields the cytosine cation with m/z 112.0531 after neutral loss of C_9_H_13_NO_4_S from the parent ion. The neutral loss of the intact homocysteine moiety is not observed.

A heteronuclear correlation experiment, ^1^H-^13^C-HMBC, determines the connectivity of individual spin systems present in UK2^15^. Key correlations in the HSQC/HMBC overlay are provided in the supplementary section (Fig. S1). The linkage of the cytosine and the didehydro ribose moiety is confirmed by the HMBC cross peak between H11/C8 at δ_1H_ 6.13 ppm and δ_13C_ 94 ppm (anomeric proton of the didehydro ribose moiety) and H3 at δ_1H_ 7.4 ppm (olefinic proton of the cytosine moiety at C5). The second correlation between the –CH_2_ groups at H6/C12 (δ_13C_ 26.9 ppm) and H4/C13 (δ_13C_ 26.5 ppm) connect the didehydro ribose to the homocysteine moiety. The chemical shifts and coupling pattern of UK1 are similar to those of UK2, suggesting closely related molecular structures.

**Figure 2:**
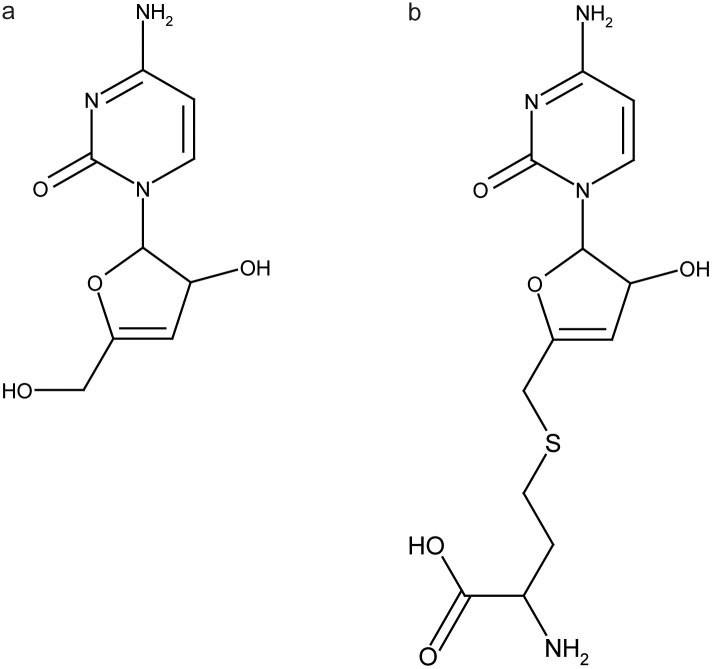
Chemical structure of UK1 and UK2. Molecular structure of 4-amino-1-[3-hydroxy-5-(hydroxymethyl)-2,3-dihydrofuran-2-yl]pyrimidin-2(1H)-one (molecular formula: C_9_H_11_N_3_O_4_; monoisotopic mass: 225.074956; chemical shift: 4.27(m), 4.96(t), 5.38(d), 6.17(d), 6.24(d), 7.60(d)) (a). Molecular structure of 2-amino-4-({[5-(4-amino-2-oxopyrimidin-1(2H)-yl)-4-hydroxy-4,5-dihydrofuran-2-yl]methyl}sulfanyl)butanoic acid (molecular formula: C_13_H_18_N_4_O_5_S; monoisotopic mass: 342.37082; chemical shift: 2.07(m), 2.61(t), 3.35(q), 3.73(t), 4.88(t), 5.24(d), 5.93(d), 6.13(d), 7.40(d)) (b).

Detailed evidence for assignment is provided in the supplement (Fig. S1). Based on the spectroscopic data the two unknown metabolites are identified as (i) 4-amino-1-[3-hydroxy-5-(hydroxymethyl)-2,3-dihydrofuran-2-yl]pyrimidin-2(1H)-one (molecular formula: C_9_H_11_N_3_O_4_; monoisotopic mass: 225.074956; chemical shift: δ 4.27(m), 4.96(t), 5.38(d), 6.17(d), 6.24(d), 7.60(d)) and (ii) 2-amino-4-({[5-(4-amino-2-oxopyrimidin-1(2H)-yl)-4-hydroxy-4,5-dihydrofuran-2-yl]methyl}sulfanyl)butanoic acid (molecular formula: C_13_H_18_N_4_O_5_S; monoisotopic mass: 342.37082; chemical shift: δ 2.07(m), 2.61(t), 3.35(q), 3.73(t), 4.88(t), 5.24(d), 5.93(d), 6.13(d), 7.40(d)). These two metabolites that are structurally identified from the combined NMR and MS information are likely to be pathway related (Fig. 2). The metabolic profiling pipeline developed to identify the two unknown potential diagnostic markers is depicted in Fig. S2.

The third unknown candidate biomarker for the *P. berghei*-infected groups is associated with doublets at δ_1H_ 1.21 and δ_1H_ 1.24, which are statistically correlated but could not be cleanly isolated. The compound does not ionize in the mass spectrometer. Since this compound only discriminates *P. berghei* infection at a single timepoint, we did not pursue identification any further. Neither pipecolic acid nor metabolites UK1 and UK2 vary significantly in urinary concentration between mice with single and dual infection, with the exception that urinary pipecolic acid levels are higher in the day 19 simultaneous co-infection group (SC) than in the single *P. berghei* infection group (P).

### Comparison of *P. berghei* single-infection with two hookworm co-infection scenarios

**Figure 3:**
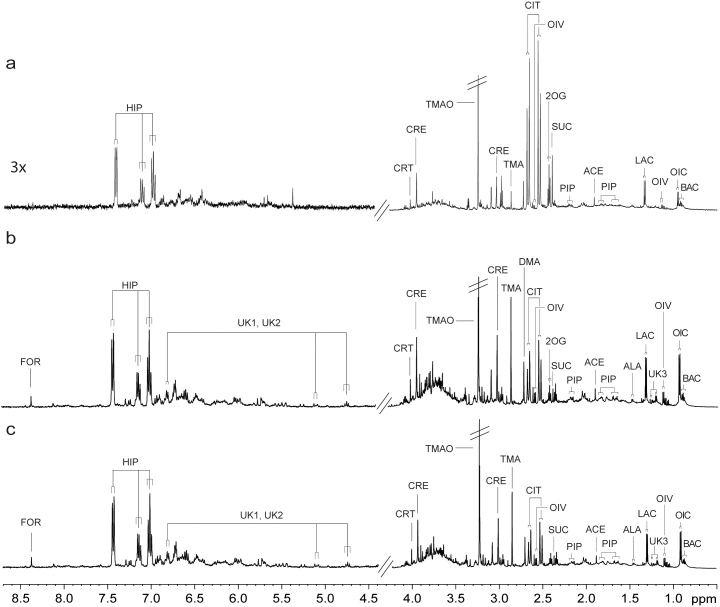
Typical ^1^H NMR-derived urine spectra. Representative sample obtained from an uninfected control mouse (a), mouse with *P. berghei* single infection (b), and mouse with a delayed *P. berghei-H. bakeri* co-infection (c). The key to metabolite identity is the one indicated in Tables 1 and S5. Additional abbreviations: ALA, alanine; CIT, citrate; CRT, creatinine; DMA, dimethylamine; 2OG, 2-oxoglutarate; TMAO, trimethylamine-*N*-oxide. The water peak region was removed and the TMAO resonance truncated (as indicated by double bars) to allow vertical expansion.

The urine and plasma spectral profiles show that all infection models specifically imprint on the host metabolism (Tables 1, S1–S3). We show a typical ^1^H NMR urine spectrum from (a) an uninfected control mouse (Ctr), (b) a *P. berghei*-infected animal (P), and (c) a mouse that underwent delayed co-infection (DC) at experimental day 19 (Fig. 3). In addition to the four unique candidate urinary biomarkers of *P. berghei*, three of which are consistently elevated across the single infection and both co-infection groups (Table 1), changes in the concentrations of several endogenous metabolites are apparent for *P. berghei* infection. In brief, urine samples collected on day 16 show a number a relative increase in acetate in mice simultaneously infected with *P. berghei* and *H. bakeri* (group SC), compared to non-infected mice (Ctr group) and an increase of 1-methylnicotinamide in the *P. berghei*-infected group (P) compared to uninfected animals (Ctr). On day 19, concordant with increasing severity of infection, additional changes such as an increase in creatine, 2-oxoisovalerate, and 2-oxoisocaproate are observed in the SC group in comparison with either control group (Ctr or H). 2-Oxoisocaproate is also elevated in the single *P. berghei* infection (P) over the delayed co-infection (DC), whilst succinate is present in relatively lower concentrations in all *P. berghei*-infected groups relative to controls.

The plasma profiles of animals infected with *P. berghei* show a marked but changing response over the course of malaria infection but do not yield any specific candidate biomarkers of *Plasmodium* infection (Supplementary information, Tables S2, S3).

### Parasite burden, body weight, and packed cell volume

Parasitemia levels do not significantly differ in any of the pairwise comparisons between the three *P. berghei*-infection groups. Levels of 39.5% (±9.4%), 35.3% (±11.7%), and 37.1% (±10.1%) are recorded for the groups P, DC, and SC, respectively. Average worm counts of 42.4 (±17.7) and 61.9 (±18.4) correspond to groups H and DC, respectively (*p* = 0.053). The worm burden cannot be assessed in the simultaneous co-infection, since *H. bakeri* larvae are too small and embedded in the intestinal mucosa. No significant change in body weight is observed between groups at any of the timepoints assessed (detailed results are provided in Table S4). Packed cell volume (PCV) values, on the other hand, are significantly lower in group P compared to group H (*p* = 0.012), and group SC (*p* = 0.019) one day before infection (day −1). However, this is no longer the case on day 1 post-infection. There is no significant variation in mean PCV values between the co-infection groups, or between *P. berghei*-infected mice and each co-infection group. Infection with *H. bakeri* alone does not lead to decreased PCV compared to group Ctr. The mean PCV is lower in group P than in group DC (*p* = 0.040) on day 16 only. Group P presents a significantly lower mean PCV value (37.7%) compared to groups H or Ctr (52.6% and 51.9%, *p* = 0.003 and 0.006, respectively) on day 19. Groups DC and SC exhibit lower PCV values than the control groups (H and Ctr), with mean PCV values of 28.5% and 34.3% (all *p* ≤ 0.01) (Table S4).

## Discussion

Candidate urinary biomarkers specific to *P. berghei* infection are characterized from both single and hookworm co-infection models of murine malaria. We find four discriminatory metabolites in the urine of *P. berghei*-infected mice using a new analytical pipeline. We deem three of these metabolites worthy for testing in human populations as candidate biomarkers for malaria: namely pipecolic acid, 4-amino-1-[3-hydroxy-5-(hydroxymethyl)-2,3-dihydrofuran-2-yl]pyrimidin-2(1H)-one (UK1), and its derivative 2-amino-4-({[5-(4-amino-2-oxopyrimidin-1(2H)-yl)-4-hydroxy-4,5-dihydrofuran-2-yl]methyl}sulfanyl)butanoic acid (UK2). These metabolites are detected in *P. berghei*-infected groups (P, DC, and SC) but not in control mice (Ctr) or animals with *H. bakeri* single-infection (H). We are not able to conclusively identify the fourth metabolite (UK3), which is part of the differential profile of all *P. berghei* infected groups versus groups H and Ctr on day 16 post-infection. Of the three structurally identified metabolites specific to *P. berghei* infection, pipecolic acid has been reported previously and also unknown doublet signals at δ 6.27 (UK1 and UK2) and δ 1.20, 1.24 (UK3) associated with *P. berghei*, were reported in the paper by Li et al. Pipecolic acid was found to be positively discriminatory for rodent malaria from day 3 postinfection onwards^9^.

Pipecolic acid has been described in human *P. vivax* infections and may hence represent a diagnostic biomarker for infection with *Plasmodium* spp.^16^. In humans, accumulation of the compound has been associated with a range of health conditions such as liver dysfunction or neurological damage^17^,^18^,^19^, whereas pipecolic acid in plants has been described as a key regulator of immunity to microbes^20^. However, the role of pipecolic acid in *Plasmodium* infection remains elusive.

4-amino-1-[3-hydroxy-5-(hydroxymethyl)-2,3-dihydrofuran-2-yl]pyrimidin-2(1H)-one, and 2-amino-4-({[5-(4-amino-2-oxopyrimidin-1(2H)-yl)-4-hydroxy-4,5-dihydrofuran-2-yl]methyl} sulfanyl)butanoic acid are consistently detected in the urine of mice from all *P. berghei*-infected groups, on days 16 and 19 and, to our knowledge, neither of these metabolites has been observed before. They might therefore be the most promising candidate biomarkers of *P. berghei* infection. There is no significant difference between the single and co-infected *P. berghei* groups in the level of excretion. These metabolites are closely-related and share a common cytosine bound to didehydro ribose as backbone structure. However, 2-amino-4-({[5-(4-amino-2-oxopyrimidin-1(2H)-yl)-4-hydroxy-4,5-dihydrofuran-2-yl]methyl}sulfanyl)butanoic acid carries an additional homocysteine.

The structural similarity between UK1 and UK2 suggests that these compounds share a pathway-related relationship. Homocysteine, which is a constituent of UK2 has been reported to be found in higher concentrations in the plasma in individuals with malaria or malnutrition^21^. The malaria parasite uses S-adenosyl-L-homocysteine (SAH) hydrolase to combat the toxicity of S-adenosyl-L-homocysteine by converting SAH into adenosine and homocysteine^22^. Free homocysteine is not observed in the NMR spectra of either urine or plasma from *P. berghei* infected groups, although the resonances may easily have been overshadowed by high concentration metabolites from macromolecules, such as the lipoproteins, in the plasma.

Formation of UK1 is likely to be brought about by enzymatic breakdown by an enzyme such as S-adenosyl-L-homocysteine hydrolase or similar to remove the homocysteine moiety from UK2. Since UK1 and UK2 have not previously been reported in the literature, their molecular origin, their biological function and subsequent metabolism is at this stage speculative. However, there is a reasonable probability of *N*-acetylation on either of the amino groups and this may influence the capacity of this molecule for protein binding.

Several studies have reported an exacerbated response to malaria following hookworm infection in humans, but we find that *P. berghei* parasitemia, PCV, and body weight did not significantly vary in the presence of *H. bakeri*, and neither did the absolute worm counts in the presence of the *P. berghei* co-infection, which supports the finding of de Souza et al. in C57Bl/6 mice^23^. A possible explanation for this observation is that the murine intestinal nematode does not induce anemia and, hence, is not representative of the stress imposed by the hookworm in the human scenario^24^,^25^. However, although the gross physiological measures suggest that no significant parasitologic interaction took place, clear differences between the infection scenarios were identified in the urine and plasma composition, which demonstrates that the complexity and specificity of host-parasite interactions can still be reflected at the metabolic level without gross differences in parasitemia.

The key outcome of this work is that the methodological framework developed here for metabolic profiling-based biomarker discovery, incorporating a sequential array of spectroscopic assays and statistical spectroscopy, has genuine potential in parasite diagnostics. Perhaps the application to parasitic infection is even more compelling than in other disease scenarios because of the co-existence of metabolites from both host and parasite. Here, two unique structurally related urinary candidate biomarkers for murine malaria are identified and, to our knowledge, have not been described so far in the eukaryotic organism. We are furthermore able to confirm the presence of previously described urinary pipecolic acid and a third unknown (UK3) in *P. berghei* single and co-infections. Our work presents here three candidate biomarkers (i.e. pipecolic acid, 4-amino-1-[3-hydroxy-5-(hydroxymethyl)-2,3-dihydrofuran-2-yl]pyrimidin-2(1H)-one and 2-amino-4-({[5-(4-amino-2-oxopyrimidin-1(2H)-yl)-4-hydroxy-4,5-dihydrofuran-2-yl]methyl}sulfanyl)butanoic acid) to take forward for validation as early and specific markers of *plasmodium* infection in human cohorts.

## Methods

### Sample origin

Details for the rodent model, infection parameters, and sampling conditions are given in the supplementary information. In brief; the current work was approved by the Swiss cantonal and national regulations of laboratory animal welfare (permission no. 2081). Forty 3-week-old female NMRI mice were split randomly into 5 groups P, H, SC, DC, and Ctr (n = 8). *Plasmodium* infection in groups P, DC, and SC was established with 2 × 10^7^ erythrocytes, parasitized with the green fluorescent protein (GFP)-transfected *P. berghei* ANKA strain in 0.2 ml red blood cell solution in RPMI medium intravenously. Groups H, DC, and SC received 80 infective *H. bakeri* third stage larvae (L_3_) which were administered orally in 150 μl water. Urine and blood were collected from all mice one day before and after each infection timepoint (1 day preinfection and days 1, 14, and 16), during the maturing helminth single infection (day 8) and four days post *P. berghei*-infection (day 19).

### Sample preparation and ^1^H nmr spectroscopic analysis

Urine samples were prepared by mixing 30 μl phosphate buffer (43.8 mM NaH_2_PO_4_ and ~0.2 M Na_2_HPO_4_, 70% D_2_O v/v, 0.1% sodium 3-(trimethylsilyl) propionate-2,2,3,3-*d*_4,_ pH = 7.4) with 30 μl urine.

The prepared samples were transferred into NMR microtubes (Bruker, diameter: 1.7 mm) shortly before measurement, and stored at 4°C prior to spectral acquisition. A standard ^1^H NMR spectrum was acquired from each individual sample on a Bruker DRX 600 MHz spectrometer (Bruker Biospin, Rheinstetten, Germany), in a standard 1D experiment, using the standard solvent suppression pulse delay [recycle delay (RD)-90°-*t*_l_-90°-*t*_m_-90°-acquire free induction decay (FID)]^26^. The relaxation delay (RD) was typically 2 s long and *t*_I_ at 3 μs, while the mixing time (*t*_m_) was set to 100 ms. Water irradiation was performed during the relaxation delay and also during the mixing time. Acquisition time for each sample was 2.73 s and spectral width was set to 20.017 p.p.m. A line broadening factor of 0.3 Hz was applied to the FID and the FIDs were Fourier-transformed into spectra of 65.5 K points resolution. The samples were scanned 256 times in each experiment at a constant temperature of 300 K. Details for plasma preparation and analysis are given in the supplementary material.

### Data reduction and multivariate analysis

All ^1^H NMR spectra were manually phased and baseline-corrected in Topspin (version 3.1, Bruker), referenced to TSP at δ 0.00. The complete spectra were imported into MATLAB (version 7.12.0, R2011a) and the water peak region was removed in all spectra (δ 4.18–5.77). Further spectral pre-processing included probabilistic quotient normalization and peak alignment, using in-house developed scripts^27^.

O-PLS-DA was applied to compare ^1^H NMR spectral data between the different murine infection groups and to identify the discriminating compounds (biomarkers)^28^,^29^. Assignment of unknowns was performed using MS and NMR data and the CMC-se software (Bruker Biospin, Rheinstetten, Germany).

### Validation of the candidate biomarkers

Candidate biomarkers identified in O-PLS-DA (p < 0.05) were further validated by univariate analysis. The area under the curve (AUC) was extracted from the clearest signal for each metabolite found significant *via* O-PLS-DA analysis. Variance analysis between the mean values of the groups was performed using a Mann-Whitney U test with Bonferroni correction, with a significance cut-off level of 5%. Peaks that were found to be statistically insignificant according to the Mann-Whitney U test were removed unless they belonged to the top five correlation coefficients extracted by multivariate (O-PLS-DA) analysis. All identified peaks that showed significance in both analysis sets were documented (Tables 1, S1–S3, and S5).

### Identification of metabolic biomarkers

Metabolite identity was determined using the literature^9^,^26^,^30^,^31^,^32^,^33^, statistical total correlation spectroscopy (STOCSY)^29^, and the software Chenomx Profiler (Chenomx NMR Suite, 7.1, evaluation version).

The structural identity of the urinary metabolic discriminators UK1 and UK2 was investigated by UPLC-TOF-MS/MS and LC-NMR/TOF-MS. The UPLC-MS results were obtained on a Waters Acquity UPLC system (Milford, MA, USA) using a 100 × 2.1 mm 1.7 μm BEH C18 column at a flow rate of 0.25 ml/min. The mobile phase consisted of acetonitrile 0.1% formic acid (B) and water 0.1% formic acid (A) with a linear solvent gradient starting with 100% A, changing to 60% A after 12 min and to 0% A at 13 min. At 13.1 min, the composition was changed back to 100% A for re-equilibration of the column. The injection volume was 5 μL. The chromatography was coupled to a micrOTOF-Q mass spectrometer from Bruker Daltonic (Bremen, Germany) operated in positive electrospray ionization mode with a scan range from 50 to 1000 m/z. MS/MS measurements were conducted at 22 eV with nitrogen as collision gas after isolation of the precursor mass with m/z 343.

The LC-NMR/TOF-MS measurements were made on an Agilent 1260 HPLC system (Waldbronn, Germany) coupled to a micrOTOF mass spectrometer (Bruker Daltonic, Bremen Germany) and an AV III 600 MHz NMR spectrometer equipped with a 5 mm BBO cryo probe (Bruker Biospin, Rheinstetten, Germany). A peak sampling unit (BPSU-36, Bruker Biospin, Rheinstetten, Germany) was used to store chromatographic peaks prior to transfer into a vial. The separation was achieved with acetonitrile 0.1% formic acid (B) and deuteriumoxide 0.1% formic acid (A) on a 250 × 4.6 mm 5 μm Agilent Zorbax C18 column using a solvent gradient changing from 100% A to 90% A in 20 min. A total volume of 40 μL of pooled infected mouse urine was injected on column. The peaks with m/z 231 (UK1, exchange of 4 protons with deuterium) and 350 (UK2, exchange of 6 protons with deuterium) were stored from 3 (UK1) and 5 (UK2) individual injections in storage loops, transferred to a vial and evaporated to dryness with a stream of nitrogen. The two compounds were re-dissolved in 600 μL deuteriumoxide and measured in the cryo probe.

1D and 2D NMR experiments were conducted to elucidate the structure of UK2: A ^1^H-^13^C-HMBC was recorded with 1024 scans in 160 increments to determine the connectivity of individual spin systems present in UK2. A ^1^H-^13^C-HSQC was obtained for the detection of proton-carbon 1J-connectivities with 128 scans in 256 increments. Individual spin systems were identified from the ^1^H-^1^H-COSY acquired with 16 scans in 256 increments. The structure of UK2 was calculated from the molecular formula and the set of NMR data using the CMC-se software from Bruker Biospin (Rheinstetten, Germany). Details for the assignment of the connectivity of individual spin systems and the MS/MS spectrum for UK2 are provided in Fig. S1. UK1 is structurally strongly related to UK2; in-depth structure elucidation was therefore not necessary. LC-MS and LC-NMR/MS. Pooled urine samples were injected on a 250 × 4.6 mm Kinetex C18 column with a particle diameter of 5 μm. The separation was done on a 1200 series Agilent HPLC system consisting of a quaternary HPLC pump, an auto sampler, a column oven and diode array detector. The mobile phase consisted of D_2_O 0.1% formic acid (A) and acetonitrile 0.1% formic acid (B). A linear gradient was applied from 100% A at 0 min to 95% A at 20 min. The flow rate was 0.8 ml/min and the injection volume 50 μl. After chromatography, 2% of the flow was split to a micrOTOF time-of-flight mass spectrometer (Bruker Daltonics, Bremen, Germany) operated in positive ionization mode. Based on UV and MS response, the peaks of interest were captured in the storage loops of a Bruker Peak Sampling Unit (BPSU, Bruker Biospin, Rheinstetten, Germany). Ten injections were collected and pooled in a vial and evaporated to dryness with a stream of nitrogen gas. After re-constitution with D_2_O, all necessary experiments (1D-Proton, COSY, HSQC and HMBC) were conducted to calculate the most probable structure using the CMC-se software package (Complete Molecular Confidence-structure elucidation software from Bruker Biospin, Rheinstetten, Germany). This software picks the resonance peaks from the NMR data acquired and populates a correlation table first. Together with the molecular formula obtained from the high resolution mass spectrometry measurement the software then calculates possible structures which are consistent with the signals in the correlation tables. Finally, the software predicts the ^13^C chemical shift values for each possible structure and compares and ranks this result with the real shifts. According to this, the most probable structures for the unknown metabolites are shown in Fig. 2.

## Supplementary Material

Supplementary Information Supplementary Material

## Figures and Tables

**Table 1:** List of metabolites found in urine during *P. berghei* single and co-infection

ID.	Metabolite	Day 16							Day 19								
		P/H	P/Ctr	H/DC	H/Ctr	H/SC	DC/Ctr	SC/Ctr	P/H	P/DC	P/Ctr	P/SC	H/DC	H/SC	DC/Ctr	DC/SC	SC/Ctr
MNA	1-methylnicotinamide																
OIC	2-oxoisocaproate																
OIV	2-oxoisovalerate																
ACE	acetate																
BAC	bile acids																
CRE	creatine																
FOR	formate																
HIP	hippurate																
LAC	lactate																
PIP	pipecolic acid																
SCY	*scyllo*-inositol																
SUC	succinate																
TMA	trimethylamine																
UK1	4-amino-1-[3-hydroxy-5-(hydroxymethyl)-2,3-dihydrofuran-2-yl]pyrimidin-2(1*H*)-one																
UK2	2-amino-4-({[5-(4-amino-2-oxopyrimidin-1(2*H*)-yl)-4-hydroxy-4,5-dihydrofuran-2-yl]methyl}sulfanyl)butanoic acid																
UK3	unknown 3																

Table 1. Urine biomarkers identified during *P. berghei* infection, specific to each single and co-infection designed. Red and black arrows refer to increase and decrease, respectively in the first group cited in each column (i.e. P in P/H). Key: P, *P. berghei* only; H, *H. bakeri* only; DC, delayed co-infection; SC, simultaneous co-infection; Ctr, uninfected control.
