# Supplementary material for: Metabolic Profiling Framework for Discovery of Candidate Diagnostic Markers of Malaria
Source: Sci Rep. 2013 Sep 26;3:2769. doi: 10.1038/srep02769 (PMC6505674; doi:10.1038/srep02769)
Supplement: Supplementary Information — Supplementary Material [file srep02769-s1.pdf]

Supplementary Information

Metabolic Profiling Framework for Discovery of Candidate Diagnostic Markers of Malaria.

Lucienne Tritten[1,2], Jennifer Keiser[1,2], Markus Godejohann[3], Jürg Utzinger[2,4], Mireille Vargas[1,2], Olaf Beckonert[5], Elaine Holmes[5], & Jasmina Saric[5]

[1]Department of Medical Parasitology and Infection Biology, Swiss Tropical and Public Health Institute, CH-4002 Basel, Switzerland. [2]University of Basel, CH-4003 Basel, Switzerland; [3]Bruker BioSpin GmbH, 76287 Rheinstetten, Germany. [4]Department of Epidemiology and Public Health, Swiss Tropical and Public Health Institute, CH-4002 Basel, Switzerland. [5]Section of Computational and Systems Medicine, Department of Surgery and Cancer, Faculty of Medicine, Imperial College, South Kensington, London, SW7 2AZ, United Kingdom. Correspondence and requests for materials should be addressed to J. S. (email: jasmina.saric@imperial.ac.uk).

Material and Methods

Parasite-Rodent Model. The current work was approved by the Swiss local and national regulations of laboratory animal welfare (permission no. 2081). Forty 3-week-old female NMRI mice (Charles River, Sulzfeld, Germany) were kept in groups of 8 in macrolon cages under standard environmentally-controlled conditions (temperature: 25°C, humidity: 70%, light/dark cycle 12/12 h). Mice had access to water and rodent food *ad libitum* (Rodent Blox from Eberle NAFAG, Gossau, Switzerland) and were acclimatized in the animal facility of the Swiss Tropical and Public Health Institute (Swiss TPH) for one week before the first sampling was conducted. Each group of eight mice was allocated to a different infection schedule (Fig. 1). On day 0, two groups received 80 infective *H. bakeri* third stage larvae (L_3) which were administered orally in 150 µl water. Three groups of mice received 2 x 10^7 erythrocytes, parasitized with the green fluorescent protein (GFP)-transfected *P. berghei* ANKA strain in 0.2 ml red blood cell solution in RPMI medium intravenously [1], on day 15. Urine and blood were collected from all mice one day before and after each infection timepoint (1 day preinfection and days 1, 14, and 16), during the maturing helminth single infection (day 8) and four days post *P. berghei*-infection (day 19). On each sampling day, mice were monitored for weight and PCV. All mice were euthanized on day 19 and parasitemia was evaluated by FACScan (Becton Dickinson; Basel, Switzerland) for *P. berghei* [1] and by counting of the worms *via* binocular (16x) after manual removal of the worms from the intestine. For PCV, weight, worm counts and *P. berghei* parasitemia, as well as inter-group median variation was analyzed using the Mann-Whitney U test with Bonferroni correction in StatsDirect (version 2.4.5; StatsDirect Ltd; Cheshire, UK), with a significance level of 5%.

Biofluid Collection, Weight and Packed cell volume. Urine (at least 40 µl) and tail blood (at least 50 µl) were collected one day before infection and 1, 8, 14, 16 and 19 days after the first infection timepoint on day 0, always between 8 and 11 AM (Fig. 1). Mice were individually placed into empty cages and monitored until they released a minimum of 40 µl urine which was immediately collected into 1.5 ml Eppendorf tubes and frozen on dry ice. All

samples were stored at -80°C prior to ^1H NMR acquisition. Approximately 50 µl tail blood was sampled from each mouse into a Na-heparinized hematocrit tube (1.55 mm Ø, BRAND GMBH + CO KG; Wertheim, Germany) and centrifuged at 11,000 x rpm for 5 min (microcentrifuge Sigma 1-15).

Sample Preparation and ^1H NMR Spectroscopic Analysis. Plasma samples were prepared by mixing 25 µl plasma with 30 µl NaCl solution (0.9% NaCl, 10% D_2O v/v, pH=7.4) in Eppendorf tubes.

The prepared samples were transferred into NMR microtubes (Bruker, diameter: 1.7 mm) shortly before measurement, and stored at 4°C prior to spectral acquisition. A standard ^1H NMR spectrum was acquired from each individual sample on a Bruker DRX 600 MHz spectrometer (Bruker Biospin, Rheinstetten, Germany), in a standard 1D experiment, using the standard solvent suppression pulse delay [recycle delay (RD)-90°-t_l-90°-t_m-90°-acquire free induction decay (FID)] [2]. The relaxation delay (RD) was typically 2 s long and t_l at 3 µs, while the mixing time (t_m) was set to 100ms. Water irradiation was performed during the relaxation delay and also during the mixing time. Acquisition time for each sample was 2.73 s and spectral width was set to 20.022 p.p.m. A line broadening factor of 0.3 Hz was applied to the free induction decay and the FIDs were Fourier-transformed into a spectral resolution of 65.5 K data points. A second set of data was acquired, using a 1D Carr-Purcell-Meiboom-Gill (CPMG) pulse [RD-90°-(τ-180°-τ)$_n$] sequence [3]. The samples were scanned 256 times in each experiment, at a constant temperature of 300 K.

Data Reduction and Multivariate Analysis. Plasma spectra were manually phased and baseline-corrected in Topspin (version 3.1, Bruker) and referenced to lactate at δ 1.33. The aliphatic region of the spectra (δ 0.5-4.6) was imported into MATLAB (version 7.12.0, R2011a) for processing and multivariate modelling in order to minimize the impact of the

water-related baseline distortion. Spectral regions containing signals from ethanol and methanol were additionally removed (δ 1.15-1.22, 3.64-3.69 and 3.355-3.375). Further spectral pre-processing included probabilistic quotient normalization and peak alignment, using in-house developed scripts [4]. The formate integral, which was the only peak found between the water peak region and δ 9.00, was tested for discriminatory power between groups, with the Mann-Whitney U test (StatsDirect).

Identification of Metabolic Biomarkers. Metabolite identity was determined using the literature [2,5-9], statistical total correlation spectroscopy (STOCSY) [10], and the software Chenomx Profiler (Chenomx NMR Suite, 7.1, evaluation version).

An enzymatic digestion with β-galactosidase (Jack Beans, Sigma) was conducted on a selected sample with high relative signals between 2.0-2.1 ppm in order to confirm the nature of the glycoprotein. A baseline sample was therefore acquired and compared to the sample immediately after addition of 0.5 U of the enzyme [6] and during the next 20 h window. The assay was run on a 400 MHz spectrometer at an ambient temperature of 300 K and at a pH of ~ 7.4. The enzyme addition resulted in cleaving off of the acetyl group resulting in a marked increase of acetate and a subsequent decrease of the 2.04 signal proving signal contribution from *N*-acetyl glycoprotein, whereby α_1-acid glycoprotein is likely to be the main contributor.

Results and Discussion

Plasma Metabolic Biomarkers. *Plasmodium* infection elicited a stronger signature in the plasma than in the urine profile in terms of the number of infection-related metabolic changes. Unlike the urine profile where endogenous changes manifested mainly at day 19, the plasma metabolite signature *P. berghei* showed a significant but inconsistent response

over the course of malaria infection (Tables S2, S3). A comparison of plasma spectra from different infection groups obtained at day 19 is provided in Fig. S3, which shows samples from: (a) an uninfected control mouse (Ctr); (b) an animal with a *P. berghei* single infection (P); and (c) a mouse with a delayed co-infection (DC).

At day 16 postinfection the co-infected groups (SC and DC) respond more to *P. berghei* infection than the single *Plasmodium* infection but by day 19 the differences between the single and co-infected *P. berghei* groups are largely resolved. However, individual metabolites demonstrate different time-dependent responses to the infection.

A relative decrease in plasma lysine concentration is found in group P compared to groups H and Ctr, whereas a relative increase in levels of the same amino acid occurs in groups H and SC, when compared to group Ctr on day 16 postinfection. Lysine can be catabolized by the gut microbiota to give rise to pipecolic acid [11]; an increase in the urinary levels of pipecolic acid was observed subsequently on day 19. Moreover, a decrease in lysine levels can also follow acute stress [12], which may be associated with the response to metabolic stress resulting from the *P. berghei* infection introduced on day 15. In addition, several amino acids such as leucine, valine, and alanine were found to be augmented in *P. berghei* single and co-infections compared to uninfected controls on day 19.

The most notable differences are however detected in glucose, alanine, glycerophosphocholine (GPC), and 3-hydroxybutyrate expression, whereby the two former metabolites are present in relatively higher concentrations in groups DC and P, and the latter two were present in relatively lower levels in the *P. berghei*-infected mice.

On day 16, glucose levels decrease in group P compared to all other groups. Interestingly, this difference is not significant by day 19. In the SC model, glucose levels are lower on day 19, compared to groups Ctr, H, and DC. Depletion of plasma glucose is one of the main findings in the *P. berghei* single infection group (P) on day 16, and is also observed in the simultaneous infection on day 19 when compared to control mice. Decreased plasma

glucose concentrations have previously been reported by Li and colleagues [5] and is consistent with the fact that *Plasmodium*-infected erythrocytes consume higher amounts of glucose than normal cells, as the parasites rely on anerobic glycolysis to obtain energy. Further manifestation of this phenomenon is the substantial increase in plasma lactate levels on day 19 in plasma in all *P. berghei*-infected groups. A relative increase of glucose is observed in group H compared to group Ctr on days 8 and 14 of the experiment and in group SC compared to group Ctr on day 14 (Table S3). Our results suggest that an established *H. bakeri* infection compensates at least in part the *P. berghei*-induced plasma glucose depletion, as shown by the lack of difference between groups DC and Ctr as well as between SC and Ctr on day 16. The literature on hookworm-related changes in blood glucose levels is inconsistent.

The current study and a previous experiment in *Necator americanus*-infected hamsters conducted by Kaul and colleagues have shown relatively higher blood sugar levels in infected animals compared to uninfected controls [13]. Furthermore, malabsorption of sugars has been demonstrated in hookworm-infected patients [14]. Similarly lower blood glucose levels were observed in *Necator americanus*-infected hamsters in a previous metabolic profiling study [15]. However, it may not be appropriate to assume a linear relationship between helminth infection, gross physiological impact, and blood glucose levels, since co-infection state, genetic background of the mice, and the resulting immune-status (e.g., degree of T cell proliferation, macrophage activation, etc.) may exert additional influence on glucose consumption and physiological distribution.

Plasma 3-hydroxybutyrate depletion was observed in groups P and DC and may be indicative of ketosis. 3-Hydroxybutyrate can be used by the brain as source of energy, when blood glucose is low [16]. Metabolic acidosis has been associated with *P. falciparum*-infection in children where accumulation of plasma hydroxybutyrate and lactate were reported [17]. We are currently unable to explain this observation, since no weight change was observed between any of the infection-groups compared to the uninfected control baseline weight but,

perhaps, as the infection progresses, the levels of 3-hydroxybutyrate are depleted by the excessive energy consumption inflicted by a *Plasmodium* infection.

On day 16, a decrease in GPC was observed in all infection groups, including single hookworm infection, compared to uninfected controls; on day 19 the same trend was observed but was less pronounced. Changes in the global lipid profile were noted in *P. berghei* infected groups on day 19 (Table S3).

Reproducibility of Biomarkers between Rodent Models. The biomarker profiles of the present *P. berghei* infection models largely agree with a previous explorative model on *P. berghei* single infection in NMRI mice [5]. Some degree of metabolic inconsistency, however, was observed between the current and previous experiments. For instance, we found no infection-related change in urinary creatinine and the set of unknown metabolites reported by Li and colleagues. Furthermore, although 2-oxoisocaproate and 2-oxoisovalerate contributed to the plasma metabolic signature of *P. berghei* in both studies, a more rapid response was observed in the study by Li *et al*. at 1 day postinfection as opposed to 4 days postinfection in the current study. The decrease in plasma GPC in all *P. berghei*-infected mice compared to uninfected control groups is however, in accordance with the results of Li and colleagues and overall changes in the blood lipid profile due to *P. falciparum* infection have also been observed by Ghosh et al. [18].

References

1 Franke-Fayard, B. *et al.* A *Plasmodium berghei* reference line that constitutively expresses GFP at a high level throughout the complete life cycle. *Mol Biochem Parasitol* **137**, 23-33 (2004).

2 Nicholson, J. K., Foxall, P. J., Spraul, M., Farrant, R. D. & Lindon, J. C. 750 MHz ^{1}H and ^{1}H-^{13}C NMR spectroscopy of human blood plasma. *Anal Chem* **67**, 793-811 (1995).

3 Meiboom, S., Gill, D. Modified spin-echo method for maesuring nuclear relaxation times. *Rev. Sci. Instrum.* **29**, 688-691 (1958).

4 Veselkov, K. A. *et al.* Optimized preprocessing of ultra-performance liquid chromatography/mass spectrometry urinary metabolic profiles for improved information recovery. *Anal Chem* **83**, 5864-5872, (2011).

5 Li, J. V. *et al.* Global metabolic responses of NMRI mice to an experimental *Plasmodium berghei* infection. *J Proteome Res* **7**, 3948-3956, (2008).

6 Bell, J. D., Brown, J. C., Nicholson, J. K. & Sadler, P. J. Assignment of resonances for 'acute-phase' glycoproteins in high resolution proton NMR spectra of human blood plasma. *FEBS Lett* **215**, 311-315, (1987).

7 Coen, M. *et al.* An integrated metabonomic investigation of acetaminophen toxicity in the mouse using NMR spectroscopy. *Chem Res Toxicol* **16**, 295-303, (2003).

8 Liu, M., Nicholson, J. K., Parkinson, J. A. & Lindon, J. C. Measurement of biomolecular diffusion coefficients in blood plasma using two-dimensional ^{1}H-^{1}H diffusion-edited total-correlation NMR spectroscopy. *Anal Chem* **69**, 1504-1509 (1997).

9 Tang, H., Wang, Y., Nicholson, J. K. & Lindon, J. C. Use of relaxation-edited one-dimensional and two dimensional nuclear magnetic resonance spectroscopy to improve detection of small metabolites in blood plasma. *Anal Biochem* **325**, 260-272, (2004).

10 Cloarec, O. *et al.* Statistical total correlation spectroscopy: an exploratory approach for latent biomarker identification from metabolic ^{1}H NMR data sets. *Anal Chem* **77**, 1282-1289, (2005).

11 Fujita, T., Hada, T. & Higashino, K. Origin of D- and L-pipecolic acid in human physiological fluids: a study of the catabolic mechanism to pipecolic acid using the lysine loading test. *Clin Chim Acta* **287**, 145-156 (1999).

12 Teague, C. R. *et al.* Metabonomic studies on the physiological effects of acute and chronic psychological stress in Sprague-Dawley rats. *J Proteome Res* **6**, 2080-2093, (2007).

13 Kaul, C. L., Talwalker, P. K., Sen, H. G. & Grewal, R. S. Changes in carbohydrate metabolism in golden hamsters infected with *Necator americanus*. *Ann Trop Med Parasitol* **76**, 475-482 (1982).

14 Falaiye, J. M., Oladapo, J. M. & Wali, S. S. Hookworm enteropathy. *J Trop Med Hyg* **77**, 211-214 (1974).

15 Wang, Y. *et al.* Systems metabolic effects of a *Necator americanus* infection in Syrian hamster. *J Proteome Res* **8**, 5442-5450, (2009).

16 Owen, O. E. *et al.* Brain metabolism during fasting. *J Clin Invest* **46**, 1589-1595, (1967).

17 Sasi, P. *et al.* Metabolic acidosis and other determinants of hemoglobin-oxygen dissociation in severe childhood *Plasmodium falciparum* malaria. *Am J Trop Med Hyg* **77**, 256-260, (2007).

18 Ghosh, S., Sengupta, A., Sharma, S. & Sonawat, H. M. Metabolic fingerprints of serum, brain, and liver are distinct for mice with cerebral and noncerebral malaria: a (1)H NMR spectroscopy-based metabonomic study. *J Proteome Res* **11**, 4992-5004, (2012).

Figures

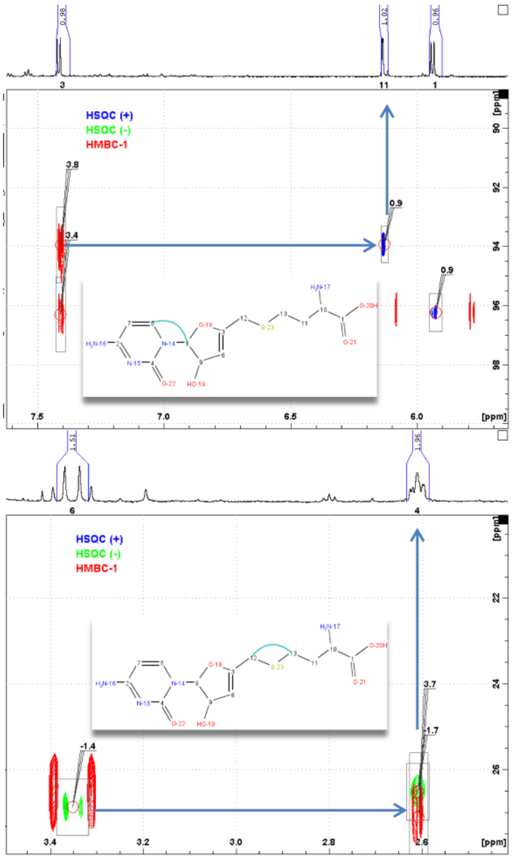

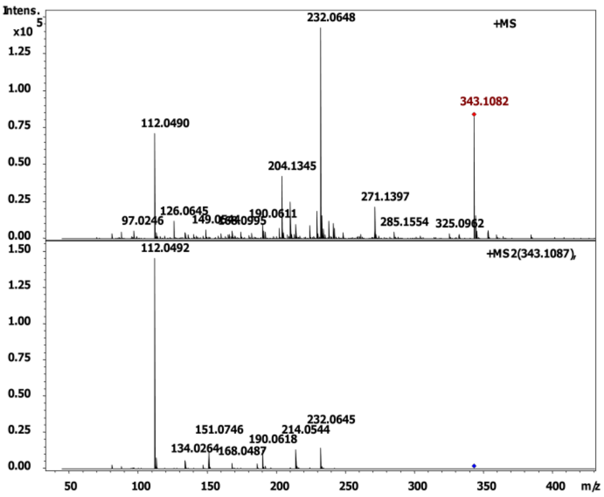

m/z	Molecular formula	Loss	Tentative assignment
112.0531	$C_4H_6N_3O$		Cytosine
232.0645	$C_7H_{12}N_4O_3S^\bullet$	$C_3H_6NO_2S^\bullet$	Cysteine radical
343.1083	$C_{13}H_{19}N_4O_5S$	$C_6H_7O_2$	

Figure S1 | Assignment details. The connectivity of individual spin systems of UK2 is determined *via* ^{1}H-^{13}C-HMBC and ^{1}H-^{13}C-HSQC spectroscopy. The MS/MS spectrum presents the main fragments of UK2.

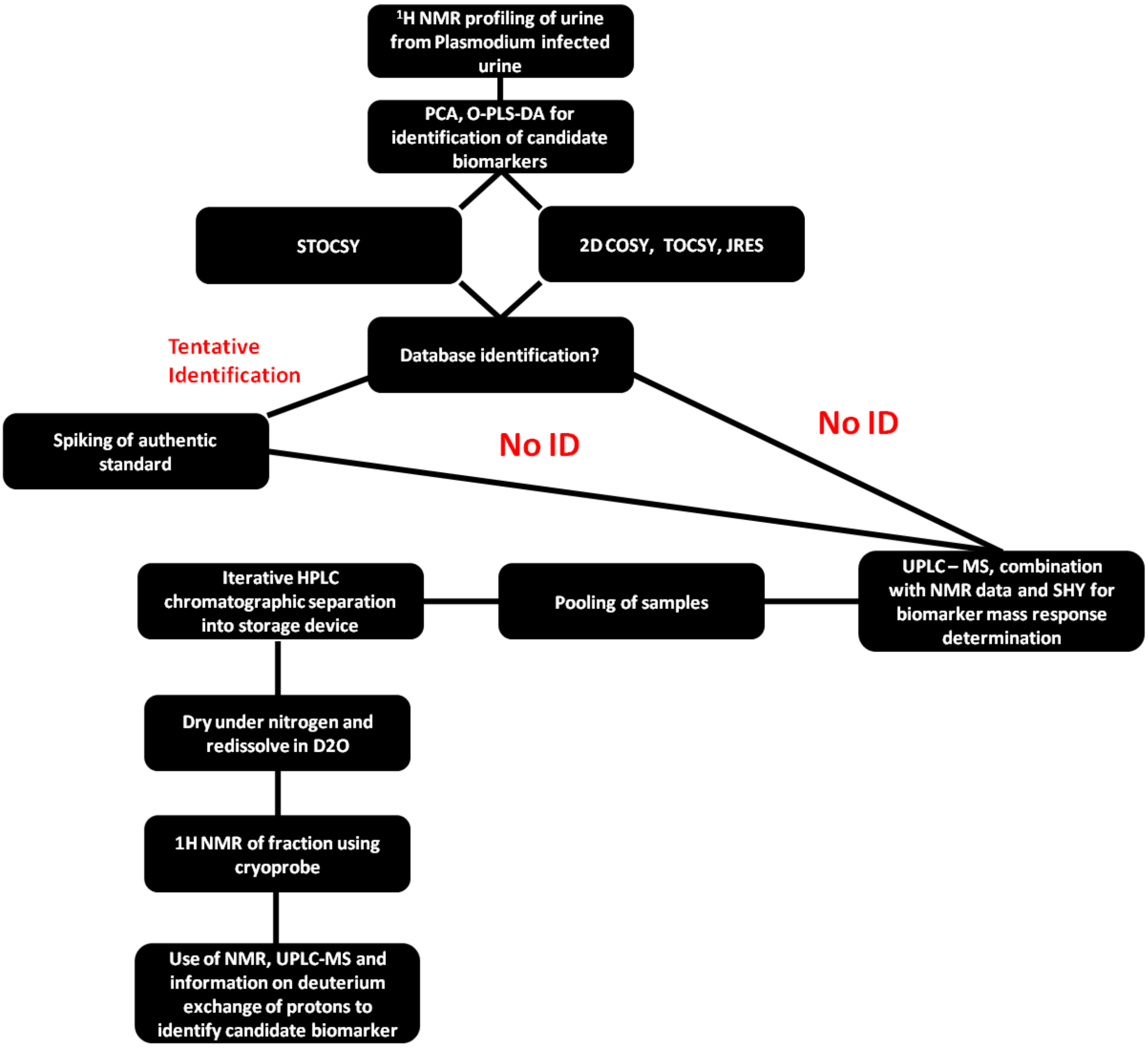

Figure S2 | Metabolic Profiling Pipeline for identification of infection-specific biomarkers.

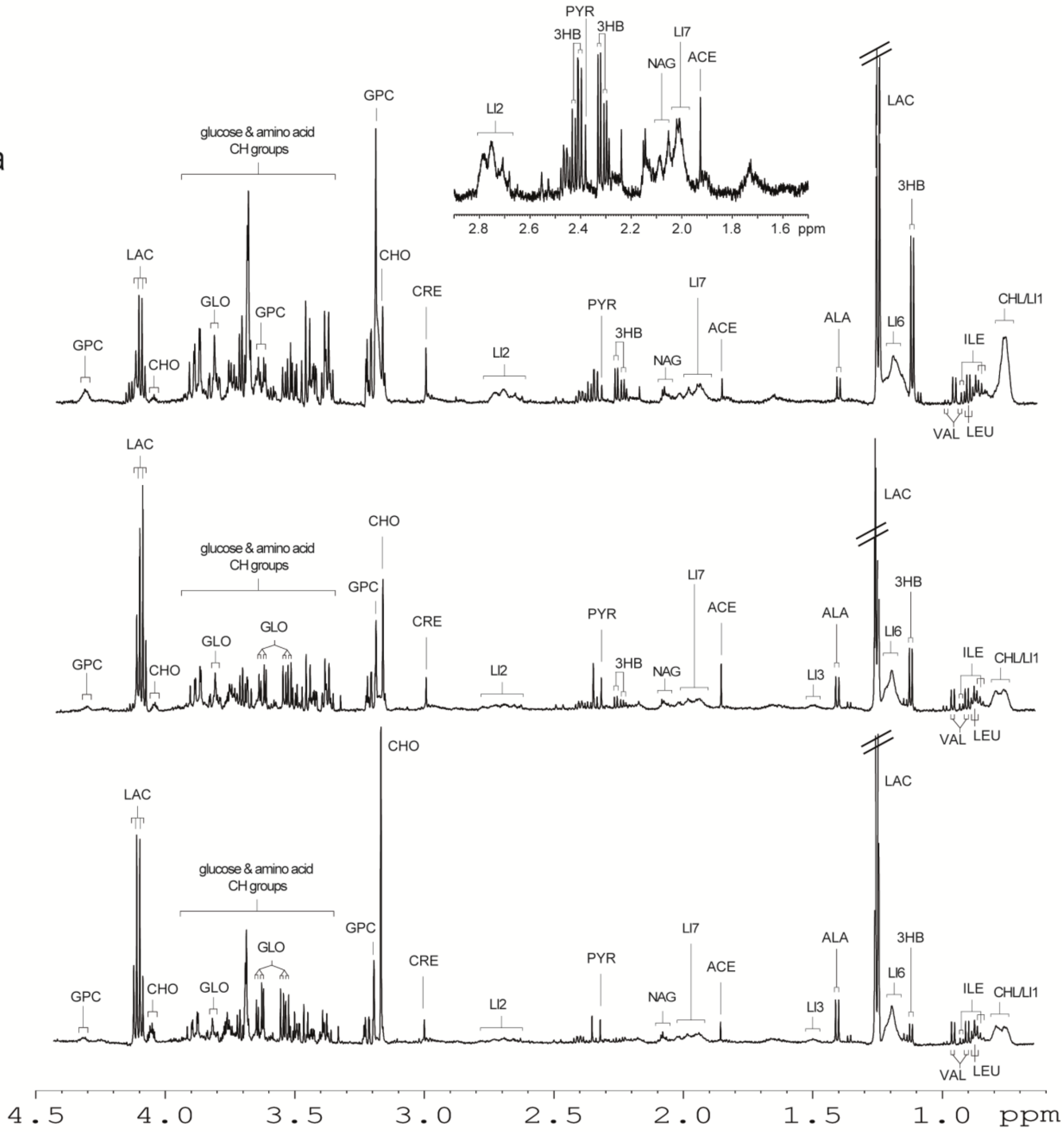

Figure S3 | Characterisation of plasma spectra. Representative ^{1}H NMR plasma spectra obtained from an uninfected control mouse (a), mouse with *P. berghei* single infection (b), and mouse with delayed co-infection (c). Only the aliphatic region is depicted due to imperfect baseline in the aromatic region. The key to metabolite identity is given in Tables S2 and S5. Additional abbreviations: LI6, $(CH_2)_n$; LI7, $CH{=}CH\mathbf{CH_2}$.

Tables

Table S1 | List of metabolic biomarkers found in urine prior to *Plasmodium berghei* infection.

**ID**	**Metabolite**	**Day -1**						**Day 1**				**Day 8**				**Day 14**							
		P/ Ctr	P/ SC	H/ DC	H/ Ctr	H/ SC	SC/ Ctr	P/ DC	P/ SC	DC/ Ctr	SC/ Ctr	P/H	P/ DC	P/ Ctr	H/ SC	P/H	P/ DC	P/ Ctr	H/ DC	H/ Ctr	H/SC	DC/Ctr	DC/ SC
**OGL**	2-oxoglutarate												↗										
**OIC**	2-oxoisocaproate		↘			↘							↗			↗					↘		↘
**OIV**	2-oxoisovalerate				↘																		
**MOV**	3-methyl-2-oxovalerate	↘	↘		↘	↘												↘					
**ACE**	acetate									↗													
**BAB**	β-aminoisobutyrate																	↘					
**CIT**	citrate			↘		↘						↘	↗	↗		↗							
**CRE**	creatine															↗	↗						
**CRT**	creatinine															↗							
**DMA**	dimethylamine									↘						↗							
**FOR**	formate																	↗				↗	
**GAC**	guanidoacetate					↘																	
**GLN**	glutamine	↘	↘																				
**GLY**	glycine			↘	↘			↗								↗							
**HIP**	hippurate							↗		↘		↗				↗					↗		
**LYS**	lysine	↗																					
**MAL**	malonate			↘	↘													↘	↘	↘			↗
**OAG**	*O*-acetylglycoprotein		↘			↘	↗																
**PHG**	phenylacetylglycine																			↘	↘		
**SCY**	*scyllo*-inositol																						↗
**SUC**	succinate							↗	↗			↘											
**TAU**	taurine	↘			↘										↘	↗	↗			↘	↘		
**TMA**	trimethylamine						↘			↘		↘						↗					
**TMAO**	trimethylamine-N-oxide		↘			↘				↘	↘					↗		↗					

Table S1. Urinary biomarkers identified prior to infection with *P. berghei*, specific to *H. bakeri*-infection (H and DC) or the different mouse groups pre-infection (Ctr, P, SC). Red and black arrows refer to increase and decrease, respectively in the first group cited in each column (i.e. P in P/H). Key: P, *P. berghei* only; H, *H. bakeri* only; DC, delayed co-infection; SC, simultaneous co-infection; Ctr, uninfected control.

Table S2 | List of metabolites found in plasma during *P. berghei* infection.

No.	Metabolite	Day 16										Day 19									
		P/H	P/DC	P/Ctr	P/SC	H/DC	H/Ctr	H/SC	DC/Ctr	DC/SC	SC/Ctr	P/H	P/DC	P/Ctr	P/SC	H/DC	H/Ctr	H/SC	DC/Ctr	DC/SC	SC/Ctr
**3HB**	3-hydroxybutyrate						↘					↘		↘		↗			↘		
**ALA**	alanine	↘		↘										↗					↗		↗
**CHL**	cholesterol		↘									↘									
**CHO**	choline	↘		↘	↘									↗	↗						
**FOR**	formate			↘					↘												
**GLU**	glucose	↘	↘	↘	↘													↗		↗	↘
**GLN**	glutamine																		↗		↘
**GLO**	glycerol						↘	↘		↘				↗			↗				↗
**GLY**	glycine				↘									↗			↗		↗		
**GLL**	glyceryl of lipids ($\mathbf{CH_2}$OCOR)		↗															↘			↗
**GPC**	glycerophosphocholine	↘		↘	↘	↗	↘	↗	↘	↘	↘	↘		↘				↗	↘		↘
**ILE**	isoleucine	↘				↘											↗				
**LAC**	lactate		↘									↗		↗		↘		↘	↗		↗
**LEU**	leucine													↗					↗		↗
**LI1**	lipid (CH_3)	↗					↘														
**LI2**	lipid ($CH_2\mathbf{CH_2}CH$)														↘	↘		↘		↘	
**LI3**	lipid ($\mathbf{CH_2}CH_2CO$)															↘		↘			↗
**LI4**	lipid ($CH_2\mathbf{CH_2}CO$)		↗	↗								↘		↗		↘		↘	↗		↗
**LI5**	lipid VLDL ($\mathbf{CH_3}CH_2CH_2C$=)	↗					↘														
**LYS**	lysine	↘		↘		↗	↗				↗										
**MYO**	*myo*-inositol	↘																			
**NAG**	*N*-acetyl glycoprotein	↗		↗														↘			↗
**PYR**	pyruvate																	↘			↗
**VAL**	valine	↘												↗					↗		↗

Table S2: Plasma biomarkers identified during *P. berghei* infection, specific to each single and co-infection designed. Red and black arrows refer to increase and decrease, respectively in the first group cited in each column (i.e. P in P/H). Key: P, *P. berghei* only; H, *H. bakeri* only; DC, delayed co-infection; SC, simultaneous co-infection; Ctr, uninfected control.

Table S3 | List of metabolites found in plasma prior to *P. berghei* infection.

ID	Metabolite	Day -1									Day 1									Day 8							Day 14									
		P/ H	P/ DC	P/ Ctr	P/ SC	H/ DC	H/ Ctr	H/ SC	DC/ SC	SC/ Ctr	P/ H	P/ DC	P/ Ctr	P/ SC	H/ DC	H/ SC	DC/ Ctr	DC/ SC	SC/ Ctr	P/ H	P/ CS	H/ DC	H/ Ctr	H/ CS	DC/ Ctr	DC/ CS	P/ H	P/ DC	P/ Ctr	P/ SC	H/ DC	H/ Ctr	H/ SC	DC/ Ctr	DC/ SC	SC/ Ctr
**HYB**	3-hydroxybutyrate																															↘				↘
**ACE**	acetate																																		↘	↗
**ACC**	acetylcarnitine							↗																												
**ALA**	alanine															↘		↘			↘				↘		↘			↘						
**CHO**	choline		↘	↘	↘	↘							↗	↗	↘		↗	↗		↘		↗	↗					↘			↘				↗	
**CRE**	creatine	↘	↘	↘	↘																															
**GLU**	glucose	↗	↗	↗				↘			↗	↗	↗			↘		↘	↗				↗									↗				↗
**GLN**	glutamine			↘			↘											↘				↗	↗	↘												
**GOL**	glycerol	↘	↘	↘				↗	↗	↘			↗									↗			↘											
**GLY**	glycine			↘							↗									↘			↗	↘												
**NAG**	*N*-acetyl-glycoprotein		↗															↘					↗					↗								
**GPC**	glycerophospho choline														↗	↗										↘										
**ILE**	isoleucine												↗													↘	↘		↗			↗	↗			
**LAC**	lactate										↘		↘																							
**LEU**	leucine								↘			↗	↗													↘	↘		↗				↗			↗
**LI1**	lipid (CH_3)																									↗		↗	↗							
**LI2**	lipid (CH_2**CH_2**CH)										↘			↘	↗																↗	↗	↗			
**LI3**	lipid (**CH_2**CH_2CO)		↗		↗																							↗								
**LI4**	lipid (CH_2**CH_2**CO)		↗		↗																							↗								
**LI5**	lipid VLDL (**CH_3**CH_2CH_2C=)				↗				↗									↘																		
**LYS**	lysine																																↗		↘	
**MYO**	*myo*-inositol	↘	↘			↘																												↘		
**PYR**	pyruvate										↘		↘	↘				↘									↘			↘						
**SUC**	succinate			↘	↘																															
**TAU**	taurine							↗																												
**VAL**	valine								↘			↗	↗													↘	↘					↗	↗			↗

Table S3. Plasma biomarkers identified prior to infection with *P. berghei*, specific to *H. bakeri*-infection (H and DC) or the different mouse groups pre-infection (Ctr, P, SC). Red and black arrows refer to increase and decrease, respectively in the first group cited in each column (i.e. P in P/H). Key: P, *P. berghei* only; H, *H. bakeri* only; DC, delayed co-infection; SC, simultaneous co-infection; Ctr, uninfected control

Table S4 | Mean weight and PCV values						
	**Day -1**	**Day 1**	**Day 8**	**Day 14**	**Day 16**	**Day 19**
**Weight in g (SD)**						
**Group P**	22.75 (1.04)	23.48 (1.28)	26.14 (1.82)	28.23 (2.05)	26.95 (2.35)	24.42 (2.06)
**Group H**	22.19 (0.69)	22.48 (0.74)	24.93 (0.92)	26.18 (1.30)	26.52 (1.59)	25.98 (1.42)
**Group DC**	22.41 (1.27)	21.90 (0.97)	24.68 (1.52)	26.02 (1.88)	25.76 (1.70)	25.71 (1.60)
**Group SC**	22.84 (2.90)	22.45 (1.27)	24.57 (1.29)	26.31 (1.59)	25.88 (1.23)	26.12 (1.24)
**Group Ctr**	22.14 (1.91)	22.63 (0.68)	24.54 (1.07)	26.42 (1.11)	26.26 (1.31)	25.67 (1.42)
**PCV in % (SD)**						
**Group P**	53.17 (4.38)	58.17 (4.04)	57.99 (3.35)	57.41 (5.33)	53.04 (2.74)	37.68 (3.70)
**Group H**	60.77 (3.18)	57.38 (4.38)	56.44 (4.93)	57.71 (3.99)	55.99 (0.89)	52.60 (3.56)
**Group DC**	56.69 (3.87)	59.74 (4.39)	59.03 (8.98)	56.94 (1.54)	57.87 (1.70)	28.46 (5.65)
**Group SC**	58.48 (1.80)	53.59 (4.04)	57.90 (5.23)	57.26 (2.98)	57.0 (7.57)	34.29 (5.70)
**Group Ctr**	55.94 (2.72)	53.43 (6.16)	57.65 (4.47)	59.51 (2.07)	55.69 (2.06)	51.90 (1.72)

Table S4. The mean weights and packed cell volumes (PCV) are given for each group (n=8).

The numbers in brackets represent the standard deviations.

Table S5 | List of metabolites identified in urine and plasma

ID	Metabolite	Chemical moiety	Chemical shift in ppm and multiplicity
ACC	acetylcarnitine	NCH_3, CH_3, ½ CH_2, ½ CH_2, ½ CH_2N, ½ CH_2N, R1-C(-**H**)(-O-CO-CH_3)-R2	3.195(s), 2.14(s), 2.65(dd), 2.51(dd), 3.85(dd), 3.61(d), 5.58(m)
ACE	acetate	CH_3	1.91(s)
ALA	alanine	CH, CH_3	3.81(q), 1.48(d)
BAB	beta aminoisobutyrate	CH_3, CH, ½ CH_2, ½ CH_2	1.2(s), 2.61(s), 3.04(dd), 3.11(dd)
BAC	bile acids	CH_3 [C-18], CH_3[C-24], CH_3[C-28]	0.72(s), 0.95(s), 0.98(d)
CHL	cholesterol	C18 (in HDL), C18 (in VLDL), C26/C27, C21	0.66(m), 0.70(m), 0.84(m), 0.91(m)
CHO	choline	3xCH_3, α-CH_2, β-CH_2	3.21(s), 4.07(m), 3.52(m)
CIT	citrate	½CH_2, ½CH_2	2.53(d), 2.66(d)
CRE	creatine	CH_3, CH_2	3.04(s), 3.93(s)
CRT	creatinine	CH_3, CH_2	3.06(s), 4.05(s)
DMA	dimethylamine	2xCH_3	2.72(s)
FOR	formate	CH	8.45(s)
GAC	guanidoacetate	CH_2	3.79(s)
GLL	glyceryl of lipids	CH_2OCOR	4.28(m)
GLN	glutamine	β -CH_2, γ-CH_2, α-CH	2.15(m), 2.46(m), 3.77 (m)
GLU	glucose	H1α, H1 β, ½ H6β, ½ H6α, H5α, ½ H6α, ½ H6β, H3α, H2α, H3β, H5β, H4α, H4β, H2β	5.23(d), 4.64(d), 3.89(dd), 3.84(m), 3.83(ddd), 3.76(m), 3.72(m), 3.71(t), 3.54(dd), 3.49(t), 3.43(ddd), 3.40(t), 3.40(t), 3.24(dd)
GLY	glycine	CH_2	3.55(s)
GLO	glycerol	½ CH_2; ½ CH_2; CH	3.56 (dd), 3.64 (dd), 3.87(m)
GPC	glycerophosphocholine	3xCH_3, ½ α-CH_2, ½ α-CH_2, ½ β-CH_2, ½ β-CH_2, γ-CH_2	3.23(s), 4.32(t), 3.60(dd), 3.68(t), 3.89(m), 3.72(dd)
HIP	hippurate	CH_2, H3/H5, H4, H2/H6	3.97(d), 7.56(t), 7.65(t), 7.84(d)
3HB	D-3-hydroxybutyrate	½ CH_2, ½ CH_2, CH, CH_3	2.32(m), 2.42(m), 4.16(tqa),1.21(d)
ILE	isoleucine	α-CH, β-CH, ½ γ-CH_2, ½ γ-CH_2, δ-CH_3, β-CH_3	3.68(d), 1.93(m), 1.25(m),1.47(m), 0.99(d), 1.02(d)
LAC	lactate	CH, CH_3	4.12(q), 1.33(d)
LEU	leucine	α-CH, β-CH_2, γ-CH, δ-CH_3, δ-CH_3	3.72(t), 1.63(m), 1.69(m), 0.91(d), 0.94(d)
LI	lipid fractions	**CH_3**CH_2CH_2C=, CH_3, $(CH_2)_n$, CH=CH**CH_2**, **CH_2**CH_2CO, CH**CH_2**CH, CH_2**CH_2**CO	0.87(t), 0.88(m), 1.25(m), 1.57(m), 2.01(m), 2.79(m), 2.23(m)
LYS	lysine	α-CH, β-CH_2, δ-CH_2, γ-CH_2, ε-CH_2	3.77(t), 1.92(m), 1.73(m), 1.47(m), 3.05(dd)
MAL	malonate	CH_2	3.11(s)
MNA	1-methylnicotinamide	H4, H3, H5, CH_3, H1	8.19(t), 8.9(d), 8.97(d), 4.48(s), 9.28 (s)
MOV	3-methyl-2-oxovalerate	terminal CH_3, CH_3, ½ CH_2, ½ CH_2, CH	0.90 (t), 1.10 (d), 1.46(m), 1.7(m), 2.93(m)
MYO	*myo*-inositol	1,3-CH, 2-CH, 5-CH, 4,6-CH	3.53(dd), 4.06(t), 3.28(t), 3.63(t)
NAG	*N*-acetyl glycoprotein	$NHCOCH_3$, *N*-acetyl CH_3	2.05 (m), 2.08 (m)
OAG	*O*-acetyl glycoprotein	*O*-acetyl CH_3	2.14 (s)
2OG	2-oxoglutarate	β-CH_2, γ-CH_2	2.44(t), 2.99(t)
OIC	2-oxoisocaproate	2xCH_3, CH, CH_2	0.93 (d), 2.09 (m), 2.62 (d)
OIV	2-oxoisovalerate	2xCH_3, CH	1.13(d), 3.02 (m)

PAG	phenylacetylglycine	δ-CH_2, α-CH_2, H2/H6, H4, H3/H5	3.67(s), 3.75(d), 7.35(m), 7.37(t), 7.42(m)
PIP	pipecolic acid	H3-H5, H3/H4, H5, H2, H2, H6	1.66(m), 1.88(m), 2.22(m), 3.04(m), 3.44(d), 3.61(dd)
PYR	pyruvate	CH_3	2.36(s)
SCY	*scyllo*-inositol	6xCH	3.35(s)
SUC	succinate	2xCH_2	2.41(s)
TAU	taurine	CH_2S, CH_2N	3.27(t), 3.43(t)
TMA	trimethylamine	3xCH_3	2.88(s)
TMAO	trimethylamine-N-oxide	3xCH_3	3.27(s)
UK1	4-amino-1-(3-hydroxy-5-hydroxymethyl-2,3-dihydrifuran-2-yl)-1H-pyrimidin-2-one	12H, 9H, 6H, 7H, 8H, 5H	4.27(m), 4.96(t), 5.38(d), 6.17(d), 6.24(d), 7.6(d)
UK2	2-amino-4-[5-(4-amino-2-oxo-2H-pyrimidin-1-yl)-4-hydroxy-4,5-dihydrofuran-2-ylmethylsulfanyl]-butyric acid	11H, 13H, 12H, 10H, 9H, 6H, 7H, 8H, 5H	2.07(m), 2.61(td), 3.35(q), 3.73(t), 4.88(t), 5.24(d), 5.93(d), 6.13(d), 7.40(d)
UK3	unknown 3	-	1.21(d), 1.24(d)
VAL	valine	α-CH, β-CH, γ-CH3, γ'-CH3	3.62(d), 2.28(m), 0.98(d), 1.03(d)

Table S5: ^{1}H NMR structural details of metabolites identified in plasma and urine, key: d, doublet; s, singlet; m, multiplet; t, triplet; dd, double doublet; q, quadruplet.
